# Opium use and risk of bladder cancer: a multi-centre case-referent study in Iran

**DOI:** 10.1093/ije/dyac031

**Published:** 2022-03-04

**Authors:** Maryam Hadji, Hamideh Rashidian, Maryam Marzban, Ahmad Naghibzadeh-Tahami, Mahin Gholipour, Elham Mohebbi, Roya Safari-Faramani, Monireh Sadat Seyyedsalehi, Bayan Hosseini, Mahdieh Bakhshi, Reza Alizadeh-Navaei, Lida Ahmadi, Abbas Rezaianzadeh, Abdolvahab Moradi, Alireza Ansari-Moghaddam, Azim Nejatizadeh, Soodabeh ShahidSales, Farshad Zohrabi, Reza Mohammadi, Mohammad Reza Nowroozi, Hossein Poustchi, Dariush Nasrollahzadeh, Farid Najafi, Ali Akbar Haghdoost, Afarin Rahimi-Movaghar, Arash Etemadi, Mohammad Ali Mohagheghi, Reza Malekzadeh, Paul Brennan, Joachim Schüz, Paolo Boffetta, Elisabete Weiderpass, Farin Kamangar, Kazem Zendehdel, Eero Pukkala

**Affiliations:** Health Sciences Unit, Faculty of Social Sciences, Tampere University, Tampere, Finland; Cancer Research Center, Cancer Institute, Tehran University of Medical Sciences, Tehran, Iran; Cancer Research Center, Cancer Institute, Tehran University of Medical Sciences, Tehran, Iran; Department of Public Health, School of Public Health, Bushehr University of Medical Science, Bushehr, Iran; Clinical Research Development Center, Persian Gulf Martyrs, Bushehr University of Medical Science, Bushehr, Iran; Social Determinants of Health Research Center, Institute for Futures Studies in Health, Kerman University of Medical Sciences, Kerman, Iran; Department of Biostatistics and Epidemiology, Kerman University of Medical Sciences, Kerman, Iran; Metabolic Disorders Research Center, Golestan University of Medical Sciences, Gorgan, Iran; Cancer Research Center, Cancer Institute, Tehran University of Medical Sciences, Tehran, Iran; Pathology and Stem Cell Research Center, Kerman University of Medical Sciences, Kerman, Iran; Research Center for Environmental Determinants of Health, School of Public Health, Kermanshah Medical Sciences University, Kermanshah, Iran; Cancer Research Center, Cancer Institute, Tehran University of Medical Sciences, Tehran, Iran; Cancer Research Center, Cancer Institute, Tehran University of Medical Sciences, Tehran, Iran; International Agency for Research on Cancer, Lyon, France; Health Promotion Research Center, Zahedan University of Medical Sciences, Zahedan, Iran; Gastrointestinal Cancer Research Center, Non-Communicable Diseases Institute, Mazandaran University of Medical Sciences, Sari, Iran; Colorectal Research Center, Shiraz University of Medical Sciences, Shiraz, Iran; Colorectal Research Center, Shiraz University of Medical Sciences, Shiraz, Iran; Golestan Research Center of Gastroenterology and Hepatology, Golestan University of Medical Sciences, Gorgan, Iran; Health Promotion Research Center, Zahedan University of Medical Sciences, Zahedan, Iran; Tobacco and Health Research Center, Hormozgan University of Medical Sciences, Bandar Abbas, Iran; Cancer Research Center, Mashhad University of Medical Sciences, Mashhad, Iran; Department of Urology, School of Medicine, Busher University of Medical Science, Bushehr, Iran; Neuroscience Research Center, Institute of Neuropharmacology, Kerman University of Medical Sciences, Kerman, Iran; Department of Urology, Kerman University of Medical Sciences, Kerman, Iran; Uro-Oncology Research Center, Tehran University of Medical Sciences, Tehran, Iran; Liver and Pancreatobiliary Diseases Research Center, Digestive Diseases Research Institute, Tehran University of Medical Sciences, Tehran, Iran; International Agency for Research on Cancer, Lyon, France; Research Center for Environmental Determinants of Health, School of Public Health, Kermanshah Medical Sciences University, Kermanshah, Iran; Social Development and Health Promotion Research Center, Kermanshah University of Medical Sciences, Kermanshah, Iran; Department of Biostatistics and Epidemiology, Kerman University of Medical Sciences, Kerman, Iran; Regional Knowledge HUB for HIV/AIDS Surveillance, Research Centre for Modelling in Health, Institute for Future Studies in Health, Kerman University of Medical Sciences, Kerman, Iran; Iranian National Center for Addiction Studies (INCAS), Tehran University of Medical Sciences, Tehran, Iran; Digestive Oncology Research Center, Digestive Diseases Research Institute, Shariati Hospital, Tehran University of Medical Sciences, Tehran, Iran; Metabolic Epidemiology Branch, Division of Cancer Epidemiology and Genetics, National Cancer Institute, Bethesda, MD, USA; Cancer Research Center, Cancer Institute, Tehran University of Medical Sciences, Tehran, Iran; Liver and Pancreatobiliary Diseases Research Center, Digestive Diseases Research Institute, Tehran University of Medical Sciences, Tehran, Iran; Digestive Oncology Research Center, Digestive Diseases Research Institute, Shariati Hospital, Tehran University of Medical Sciences, Tehran, Iran; International Agency for Research on Cancer, Lyon, France; International Agency for Research on Cancer, Lyon, France; Stony Brook Cancer Center, Stony Brook University, Stony Brook, NY, USA; Department of Medical and Surgical Sciences, University of Bologna, Bologna, Italy; International Agency for Research on Cancer, Lyon, France; Department of Biology, School of Computer, Mathematical, and Natural Sciences, Morgan State University, Baltimore, MD, USA; Cancer Research Center, Cancer Institute, Tehran University of Medical Sciences, Tehran, Iran; Cancer Biology Research Center, Cancer Institute, Tehran University of Medical Sciences, Tehran, Iran; Health Sciences Unit, Faculty of Social Sciences, Tampere University, Tampere, Finland; Finnish Cancer Registry, Institute for Statistical and Epidemiological Cancer Research, Helsinki, Finland

**Keywords:** IROPICAN, opium, bladder cancer

## Abstract

**Background:**

Bladder cancer (BC) is the 10th most common type of cancer worldwide and the fourth most common type of cancer in Iran. Opium use is considered as one of the risk factors for BC. We aim to assess the association between various parameters of opium use, which in Iran is mainly ingested or smoked in various forms, and the risk of BC.

**Method:**

In this multi-centre case-referent study in Iran, 717 BC cases and 3477 referents were recruited to the study from May 2017 until July 2020. Detailed histories of opium use (duration, amount, frequency) and potential confounders were collected by trained interviewers. Multivariable unconditional logistic regression models were used to measure adjusted odds ratio (OR) and 95% confidence intervals (CI). The ORs were adjusted for age, gender, place of residence and pack-years of cigarette smoking.

**Results:**

Regular opium consumption was associated with an increased risk of BC (OR 3.5, 95% CI: 2.8, 4.3) compared with subjects who never used opium. Compared with continuous users, the risk decreased to one-third for those who stopped opium more than 10 years ago. The adjusted OR for those who used both crude opium (teriak) and opium juice was 7.4 (95% CI: 4.1, 13.3). There was a joint effect of opium and tobacco (OR for users of both opium and tobacco 7.7, 95% CI: 6.0, 9.7).

**Conclusions:**

Regular opium use is associated with an approximately 4-fold risk for BC. The OR decreases along with the increasing time since stopping opium use.

Key Messages

- To our knowledge, this is the first large-scale case study investigating the relationship between opium use and bladder cancer.
- There was a substantial decrease in bladder cancer risk after stopping opium use.
- There was an additive interaction between opium and tobacco use.

## Introduction

Bladder cancer (BC) is the 10th most common type of cancer worldwide^1^ and the 4th most common cancer among men in Iran,\\\ with an estimated age-standardized (World Standard) incidence rate (ASR) of 14.3/100 000 in 2020.^2^ An increasing number of incident cases of BC is projected for Iran, due to ageing and population growth and lifestyle changes.^3^ Tobacco use and occupational exposure to chemical substances (e.g. metalworking fluids, diesel exhaust, polycyclic aromatic hydrocarbons, benzidine^4^) are the most important risk factors for BC.^1^^,^^5^

Opium, a highly addictive substance obtained from the unripe seedpod of the poppy plant, is illicitly consumed by millions of people worldwide, particularly in the Middle East and South Asia.^6^ Freshly taken from the poppy plant, opium contains alkaloids (e.g. morphine, codeine and thebaine). In these countries, it is often minimally processed by heating, boiling and drying and is variably adulterated with some chemicals (e.g. lead or chromium) before it reaches consumers. In this minimally processed form, opium may be consumed as crude opium (teriak), opium sap (shireh) or opium dross (sukhteh).^7^ These forms of opium may be ingested or smoked. Therefore, similar to tobacco, opium is a complex substance with many chemicals.

In recent systematic reviews,^8^^,^^9^ opium use was suggested as a potential risk factor for BC. However, the risk of under-reporting and detection bias in these studies was high.^8^^,^^9^ Moreover, some of the included studies suffered from methodological limitations, including lack of controlling for confounding variables (age, sex, cigarette smoking), small samples size and lack of information about starting age of opium use, duration of use, dose and route of consumption. A recent study about the association between opium use and BC in one of the provinces in Iran suggested that regular use of opium had been more common among BC patients than among people in their neighbourhood.^10^ None of the previous studies investigated the effect of time since stopping opium use, nor the dose-response relationship between opium use and BC risk. An International Agency for Research on Cancer (IARC) Working Group in 2020 concluded that opium use has a carcinogenic effect on humans, based on sufficient evidence of carcinogenicity in humans.^11^ BC is one of the cancers which has been shown to have a positive association with opium consumption.

In the present large-scale study, we report the association between various parameters of opium use and the risk of BC.

## Methods

The IROPICAN case-referent study was launched in 10 provinces in Iran to assess the association between opium consumption and risk of cancers of the lung, colorectum, bladder, and head and neck, compared with a joint group of referents who were frequency-matched by gender, age and place of reference with cancer cases of all four cancer types combined. These provinces were selected because the prevalence of opium use was relatively high, and also access to referral hospitals was possible. The referents were enrolled concurrently with the cases among the relatives or friends of patients from non-oncology wards or others who visited the hospital for reasons other than receiving treatment. The referents had to be free of cancer at the date of recruitment. Details of the study have been described elsewhere.^12^

For the current study, we use data of histologically confirmed primary BCs (ICD-O: C67) admitted to referral hospitals, who were recruited as cases from May 2017 until July 2020,^12^ and the pool of all referents of the IROPICAN study. The mean age at recruitment was 63.6 years for the cases and 57.4 years for the referents. All BCs were incident cases diagnosed less than 1 year before the interview.

Altogether 717 BC cases and 3477 referents were recruited to the study. Out of the cases, 587 (81.9%) were urothelial carcinomas and 130 (18.1%) were BC cases of other and unknown histology. The characteristics of the cases and controls are given in Table 1. The non-response rate among the cases was 1% and among the referents 11%, with the main reasons for non-participation including sickness and lethargy among cancer patients and lack of time or unwillingness to donate a biological sample among referents.^12^

**Table 1:** Distribution of demographic characteristics and habits of the bladder cancer cases and referents at the time of interview in Iran from May 2017 to July 2020

Variable	Bladder cancer cases	Referents
	Number (%)	Number (%)
Total	717 (100)	3477 (100)
Age		
30–39	14 (2.0)	257 (7.4)
40–49	50 (7.0)	559 (16.1)
50–59	181 (25.2)	1070 (30.8)
60–69	267 (37.2)	1092 (31.4)
≥70	205 (28.6)	499 (14.4)
Gender		
Female	93 (13.0)	1077 (31.0)
Male	624 (87.0)	2400 (69.0)
Place of residence		
Capital city of the region	267 (37.2)	1310 (37.7)
Other	450 (62.8)	2167 (62.3)
Province		
Tehran	139 (19.4)	816 (23.5)
Fars	166 (23.2)	943 (27.1)
Kerman	150 (20.9)	525 (15.1)
Golestan	46 (6.4)	374 (10.8)
Mazandaran	24 (3.4)	136 (3.9)
Kermanshah	52 (7.3)	251(7.2)
Khorasan-Razavi	30 (4.2)	170 (4.9)
Bushehr	56 (7.8)	84 (2.4)
Hormozgan	27 (3.8)	78 (2.2)
Systan-Balouchestan	27 (3.8)	100 (2.9)
Occupation		
High-skilled white-collar	202 (28.2)	1011 (29.1)
Low-skilled white-collar	153 (21.3)	575 (16.5)
High-skilled blue-collar	273 (38.1)	966 (27.8)
Low-skilled blue-collar	89 (12.4)	925 (26.6)
Cigarette smoking (pack-years)		
Non-smoker	287 (40.0)	2500 (71.9)
<15	111 (15.5)	449 (12.9)
15–31	120 (16.7)	255 (7.3)
>31	184 (25.7)	229 (6.6)
Unknown	15 (2.1)	44 (1.3)

### Exposure assessment

Detailed histories of opium use among both cases and referents were collected, including duration of use, starting and stopping ages, amount, and frequency of opium use per day, week and month. The amount of opium use was asked in local units of opium use and converted to grams. Other information collected included type of opium (crude opium, opium juice and both types) and routes of administration (only smoking, only ingestion and both routes). Information on the amount of opium use at a time (in grams) and number of times per day (frequency) was also collected. All these metrics were answered for up to five separate periods of opium use, and the durations of these periods were used as weights in the calculation of weighted averages.

In the statistical analyses, ever-use of opium was defined as using opium at least once during a lifetime, and regular opium use was defined as using opium at least once a week for at least 6 consecutive months. The cumulative amount of lifetime opium use was defined as the total duration of opium use (days) multiplied by the average daily amount, which was the product of an average amount of opium used at a time and the average daily frequency of opium use. We used a 3-year lag time, which means that opium consumption during the 3 past years before the interview date was excluded.

Ever-use of cigarettes and tobacco (waterpipe, Chopogh, Nass and pipe) was defined as using any at least once during a lifetime. Regular cigarette smoking and tobacco use were defined as using any at least once a week for at least 6 consecutive months. Also, cigarette smoking was defined as light (<14 pack-years), moderate (14–20) and heavy (>20). Furthermore, occupation was defined as high- or low-skilled white-collar, high- or low-skilled blue-collar.

### Statistical analyses

Unconditional logistic regression models were used to measure adjusted odds ratios (OR) and 95% confidence intervals (CI). The ORs were adjusted for age, gender, province and pack-years of cigarette smoking. Occupation was dropped from the final models because this variable did not improve the model fit (*P* >0.2). In all analyses, non-users of opium were considered as the reference group. All statistical analyses were conducted using Stata, version 16 (Stata Corp., College Station, TX, licensed to Tampere University).

## Results

Regular opium use was more than 3-fold among BC cases than among referents (adjusted OR 3.4, 95% CI: 2.7, 4.3; Table 2). The OR of regular opium use for bladder cancer of urothelial histology was 3.5 (95% CI: 2.7, 4.4) and for other or unknown histology 2.3 (95% CI: 1.2, 4.3).

**Table 2:** Characteristics of opium use among regular opium users, and the odds ratios with opium use for bladder cancer in Iran from May 2017 to July 2020, from models including age, gender, province, cigarette pack-years. Lag 3 years

Metric of opium use	Bladder cancer cases	Referents	Adjusted odds ratio (95% CI)
	Number (%)	Number (%)	
Opium use			
Non-user	387 (54.0)	2881 (82.9)	Ref.
Irregular	27 (3.8)	135 (3.9)	1.1 (0.7, 1.8)
Regular^a^	303 (42.0)	461 (13.3)	3.4 (2.7, 4.3)
Type of opium used			
Crude opium (teriak)	251 (35.0)	405 (11.7)	3.2 (2.5, 4.0)
Opium juice (shireh)	20 (2.8)	32 (0.9)	3.8 (2.0, 7.0)
Both types	32 (4.5)	24 (0.7)	7.4 (4.1, 13.3)
Route of opium use			
Only smoking	209 (29.2)	383 (11.0)	2.8 (2.2, 3.6)
Only ingestion	27 (3.8)	30 (0.9)	6.3 (3.6 11.3)
Both routes	65 (9.1)	45 (1.3)	6.9 (4.5, 10.8)
Unknown	2 (0.3)	3 (0.1)	
Count per day^b^			
<1	141 (19.7)	345 (9.9)	2.1 (1.6, 2.8)
1–2	108 (15.1)	81 (2.3)	7.5 (5.3, 10.8)
>2	54 (7.5)	35 (1.0)	9.5 (5.8, 15.4)
Average of opium use at a time (g)			
<1	153 (21.3)	207 (6.0)	3.8 (2.9, 5.1)
1–2	62 (8.7)	146 (4.2)	2.3 (1.6, 3.3)
>2	88 (12.3)	108 (3.1)	4.1 (2.9, 5.9)
Daily dose of opium (g)^c^			
<1	141 (19.7)	268 (7.7)	2.7 (2.1, 3.6)
1–2	54 (7.5)	84 (2.4)	3.5 (2.3, 5.2)
>2	108 (15.1)	109 (3.1)	5.3 (3.8, 7.4)
Starting age of opium use			
<20	21 (2.9)	53 (1.5)	2.5 (1.4, 4.5)
20–29	89 (12.4)	143 (4.1)	3.3 (2.4, 4.7)
30–39	105 (14.6)	112 (3.2)	5.1 (3.6, 7.1)
≥40	88 (12.3)	153 (4.4)	2.8 (2.0, 3.8)
Time since stopping opium use (years)			
Current user	197 (27.5)	223 (6.4)	4.8 (3.7, 6.3)
<10	74 (10.3)	138 (4.0)	3.0 (2.1, 4.2)
≥10	31 (4.3)	93 (2.7)	1.5 (1.0, 2.4)
Unknown	1 (0.1)	7 (0.2)	–
Duration of opium use (years)			
< 9	87 (12.1)	223 (6.4)	2.6 (1.9, 3.5)
19–29	106 (14.8)	131 (3.8)	4.5 (3.3, 6.2)
>29	110 (15.3)	107 (3.1)	3.8 (2.7, 5.3)
Cumulative amount of opium (kg)^d^			
<4	96 (13.4)	229 (6.6)	2.3 (1.7, 3.1)
4–14	87 (12.1)	115 (3.3)	4.1 (2.9, 5.8)
>14	117 (16.3)	114 (3.3)	5.2 (3.7, 7.2)
Unknown	3 (0.4)	3 (0.4)	–
Cumulative count of opium use (times)^e^			
<4900	64 (8.9)	230 (6.6)	1.6 (1.1, 2.2)
4900–11 000	77 (10.7)	116 (3.3)	3.8 (2.7, 5.4)
>11000	161 (22.5)	114 (3.3)	6.8 (5.0, 9.3)
Unknown	1 (0.1)	1 (0.0)	–

a Regular opium use: using opium at least once a week for at least a 6-month consecutive period during the lifetime.

b Duration-weighted average of the period-specific daily frequencies of opium use.

c Count per day multiplied by the average of opium use at a time (g).

d Cumulative amount: the average daily amount of opium multiplied by the total duration of opium use (days).

e Cumulative count: average count per day multiplied by the total duration of opium use (days).

The OR for those who used both teriak and shireh was 7.4 (95% CI: 4.1, 13.3) compared with non-users. Ingestion of opium was more strongly associated with an increased risk of BC than smoking of opium. Moreover, in a model adjusted with the duration of opium use, the OR for those who applied the ingestion route of opium use showed a strong association, with an OR of 6.8 (95% CI: 3.6, 13.6).

Those with a cumulative consumption of less than 4 kg opium during their life had a 2.3-fold risk of BC (95% CI: 1.7, 3.1), and the OR increased to 5.2 (95% CI: 3.7, 5.3) among those who had used more than a 16-kg cumulative amount of opium.

The duration of regular opium use was moderately associated with the risk of BC. The duration of fewer than 19 years showed an OR of 2.6 (95% CI: 1.9, 3.5) and the ORs for longer duration categories were ∼4.5 (Table 2).

The average amount of opium used each time did not markedly affect the BC risk, but the frequency of daily opium use was highly associated with an increased risk of BC (Table 2). Those who used opium less than once per day had an OR of 2.1 (95% CI: 1.6, 2.8) whereas those who used opium more than two times per day had an OR of 9.5 (95% CI: 5.8, 15.4). This effect was also reflected in the OR for a lifelong cumulative count of opium use. Those who had used opium more than 11 000 times had an OR of 6.8 (95% CI: 5.0, 9.3).

The OR did not have a consistent association with the age of starting opium use. The risk was highest among those who started at the age of 30–39 years (Table 2). The risk of BC among current opium users was 4.8 (95% CI: 3.7, 6.3) but the OR dropped to 1.5 (95% CI: 1.0, 2.4) among those who had stopped opium use more than 10 years before the date of interview (Table 2). In a model adjusted with cumulative opium use after further adjustment of the previous analysis for the cumulative amount of opium use, the OR for those who had stopped opium use more than 10 years before the interview date was 0.3 (95% CI: 0.2, 0.4) and for those who had stopped opium use less than 10 years before index date was 0.5 (95% CI: 0.4, 0.7) as compared with those who still used opium at the index date (results not shown in the tables).

In the model including age, gender, province and opium use, the OR for cigarette smokers with less than 14 pack-years was 1.8 (95% CI: 1.4, 2.4), for those with 14–20 pack-years was 2.9 (95% CI: 2.2, 3.8) and for those with more than 20 pack-years was 1.2 (95% CI: 0.5, 2.6), compared with non-smokers.

The results presented above and shown in Table 2 are for males and females combined. The OR for regular opium use among females was 2.9 (95% CI: 1.0, 8.2) and among males 3.4 (95% CI: 2.7, 4.4). Because there were only 93 BC cases among women, the data do not allow study of the effects of specific measures of opium use for women.

The adjusted OR for those who used both opium and tobacco was 7.7 (95% CI: 6.0, 9.7), as compared with those who did not use tobacco or opium (Table 3). When the analysis was restricted to smoking opium only, the respective OR was 7.4 (95% CI: 5.6, 9.7).

**Table 3:** Odds ratios of the interaction of regular opium use and tobacco use (cigarette, water pipe, pipe, chewing tobacco, Chopogh) to the risk of bladder cancer in Iran from May 2017 to July 2020, adjusted for age, gender, and province. Lag 3 years

Tobacco use^a^	Opium use^a^			
	Never		Regular	
	Cases/referents	OR (95% CI)	Cases/referents	OR (95% CI)
Never	171/1951	Ref.	40/101	3.8 (2.5, 5.7)
Regular	197/725	2.2 (1.7, 2.7)	259/349	7.7 (6.0, 9.7)^b^

a Results for irregular opium use (28 cases, 135 referents) and irregular tobacco use (23 cases, 228 referents) not shown.

b Relative excess risk due to interaction: 2.7 (95% CI: 0.7, 4.7), attributable proportion due to interaction 0.4 (0.1, 0.6), synergy index 1.7 (1.1, 2.6).

## Discussion

In this large multicentre case-referent study, regular opium use was associated with an approximately 4-fold risk for BC compared with the subjects who never used opium. The OR was similar for those with confirmed urothelial histology and for those with other or unknown histology, and similar for males and females. Those who used both crude opium and opium juice had a 7-fold risk of BC. Ingested opium carried a higher risk of BC than smoked opium. The risk also increased if the duration of opium was more than 17 years or cumulative use was more than 4 kg. The risk increased along with increasing frequency of daily usage, but the average amount of opium used each time did not have much effect. The starting age of opium use did not have a major independent role in the BC risk.

Few studies, mostly consisting of small-sized case-referent studies, have evaluated the effect of opium use on the risk of BC.^8^^,^^11^^,^^13–15^ Our results showed that opium consumption increases the risk of BC by 3-fold as compared with those who have not used opium (OR: 3.4, 95% CI: 2.7, 4.3), which concurs with results of previous case-control studies on opium and BC. Ghadimi *et al.*^14^ showed that opium use was associated with an increased risk of BC with an OR of 5.0 (95% CI: 1.1, 2.3). Akbari *et al.*^15^ reported an OR of 3.9 (95% CI: 1.2, 12). A systematic review also showed that opium use was associated with an increased risk of BC compared with non-users, with a pooled OR of 3.9 (95% CI: 3.1, 4.9).^8^ Another systematic review of opium as a carcinogen showed that the pooled OR based on fixed effect model analysis was 4.1 (95% CI: 3.2, 5.1) and based on random effect model analysis was 3.8 (95% CI: 2.7, 5.4).^9^ A recent case-control study from Kerman province in Iran compared opium use in BC patients diagnosed 2013–15—i.e. slightly earlier than the cases of our study (2016–20)—with neighbourhood controls, and observed an OR of 4.4 (95% CI: 2.9, 6.5) for regular opium use, which is similar to the OR seen in our study despite differences in control selection and analysis methods.^10^ Our result restricted to urothelial BC is in line with the study conducted by Zeighami *et al.*^16^ who showed that ever-use of opium use was more common among urothelial BC cases than among referents (OR: 3.0, 95% CI: 1.6, 5.4).

The carcinogenicity mechanism of opium is not completely clear. The IARC working group found strong evidence that opium dross and opium pyrolysates exhibit characteristics of carcinogens.^11^ Another explanation is that opium use promotes tumorigenesis by influencing angiogenesis and immunosuppression and by facilitating cancer cell proliferation.^17–19^ Furthermore, the exposure of the bladder to carcinogens will increase because alkaloids in opium cause urinary retention and cystitis.^20^

Our study suggested that opium consumption by smoking carried a lower risk of BC than the ingestion route. Additionally, after considering the duration of opium use, the ingestion route still showed a higher risk of BC, which is in line with the study by Sheikh *et al.*^13^ which showed an OR of 2.6 (95% CI: 1.2, 5.4) for the smoking route and 3.8 (95% CI: 1.6, 8.9) for the ingestion route of opium use.

A novel finding of this study was the strongly decreased risk of BC for those who had stopped opium use more than 10 years before the index date as compared with those who had used the same amount but had not stopped. To our knowledge, this is the first study reporting such an observation.

We observed an additive interaction effect between opium use and tobacco. Consistently with previous studies,^21^^,^^22^ the risk of BC for tobacco alone was 2-fold but 7-fold for regular users of both opium and tobacco. There is one earlier study suggesting a joint effect of opium and cigarette smoking on the risk of BC.^22^ In that study, the interaction was multiplicative but based on only one BC case who had used opium only.

It is possible that some persons have started opium use because of pain related to symptoms of BC. Because we used a 3-year lag period in our analyses, i.e. opium use during the last 3 years before the interview was not counted, our results should be free of reverse causality bias. Even without such lag assumption, the risk of reverse causality would be small because there were only 11 cases and 17 referents who started opium use less than 3 years before the interview. Most of the opium users among both cases and referents had been using opium for more than 20 years.

A major challenge in observational studies on the effect of opium use is to collect reliable data among both cases and referents, because opium use is a stigmatized and criminal offence. This might cause misclassification bias. However, it was shown in previous studies that the sensitivity of self-reporting of opium use among cases and referents was similar.^12^^,^^23^

Our study had several strengths such as a large sample size, histological confirmation for all BC cases, and use of healthy hospital visitor referents, unlike other hospital-based case-control studies in which the referents had other diseases.^23^ The data quality in our study is high because data were collected by trained interviewers using a validated questionnaire.^12^ Due to access to detailed data on the amount of opium over time, we were able to examine the dose-response association of opium use and BC as well the effects of timing of opium use. We also had detailed information on the main confounder, i.e. tobacco, which was included in the statistical models. The response rate among both BC patents and referents was high. Although we were able to control for several potential confounders, the effect of unknown or unmeasured confounders or the residual confounding of those measured cannot be neglected.

In conclusion, the risk of BC was higher among those who were regular opium users than among those who had never used opium, with evidence of a dose-response association with frequency and cumulative amount of use. The risk decreased after 10 years following stopping the use of opium. These results are in agreement with the IARC monograph volume 126, September 2020,^11^ indicating a causal association between opium use on different types of cancers, including BC. Our study has important implications for public health practice and policy making, not only in Iran but also among opium users in other countries.

## Disclaimer

Where authors are identified as personnel of the International Agency for Research on Cancer/World Health Organization, the authors alone are responsible for the views expressed in this article and they do not necessarily represent the decisions, policy or views of the International Agency for Research on Cancer/World Health Organization.

## Ethics approval

The study was approved by the Ethics Committee of the National Institute of Medical Research Development (NIMAD) (Code: IR.NIMAD.REC.1394.027). All participants signed written informed consent to participation in the study.

## Data availability

The data underlying this article cannot be shared publicly due to privacy of individuals who participated in the study. Data may be shared on reasonable request to the corresponding author.

## Author contributions

M.H., H.R., M.M., M.G.H. did the literature review. H.R., A.N., M.G.H., M.M., E.M., R.S., M.S., B.H., M.B., R.A., V.A., S.S.H., A.N., F.N., A.M., A.R. contributed to data collection. Also, F.Z., R.M., M.N. provided clinical consultation. H.P., A.R., R.M., P.B., E.W., F.K., K.Z., E.P. designed the study. M.H., E.P. did the data analysis, interpreted data and prepared the manuscript draft. All authors critically appraised the drafts of the manuscripts and approved the final version. K.Z. is the guarantor of the study and E.P. is the senior author of the manuscript.

## Funding

The IROPICAN study was funded by the National Institute for Medical Research Development (NIMAD), Iran (grant number: 940045).
